# Plausible reasons for the resurgence of Mpox (formerly Monkeypox): an overview

**DOI:** 10.1186/s40794-023-00209-6

**Published:** 2023-12-25

**Authors:** Arghavan Zebardast, Tayebeh Latifi, Nazanin-Zahra Shafiei-Jandaghi, Mehdi Gholami Barzoki, Somayeh Shatizadeh Malekshahi

**Affiliations:** 1 Virology Department, School of Public Health, Tehran University of Medical Sciences, Tehran, Iran; 2 Department of Virology, Faculty of Medical Sciences, Tarbiat Modares University, Tehran, Iran

**Keywords:** Mpox, Orthopoxvirus, Emerging disease, Infection, Virus, Epidemic

## Abstract

Poxviruses are large and diversified viruses that cause an emerging zoonotic disease known as monkeypox (mpox). In the past, mpox predominated primarily in the rural rainforests of Central and West Africa. Recently, the exportation of mpoxv from Africa to other continents has been progressively reported. However, the lack of travel history to Africa in most of the currently reported cases in 2022 promotes the sign of changing epidemiology of this disease. Concerns over the geographic distribution and continued resurgence of mpox is growing. In this review, we addressed the geographic distribution, transmission, reasons for the resurgence of mpox, and vaccination. Although the precise cause of the resurgence in mpox cases is mostly unknown, several suggested factors are believed to be waning immunity, accumulation of unvaccinated people, ecological conditions, risk behaviors of men who have sex with men, and genetic evolution.

## Introduction

### Virological and biological features

Poxviruses are large and diversified viruses composed of a 200 kb linear double-stranded DNA [1–3]. The monkeypox virus (mpoxv) is a member of the *Poxviridae* family, chordopoxvirinae subfamily, and orthopoxvirus (opxv) genus [4]. The opxv genus has three other human-pathogenic species: variola virus (the etiologic agent of smallpox), cowpox virus, and vaccinia virus [5]. Since smallpox was eradicated in 1980, mpoxv has been regarded as the opxv posing the greatest threat to human populations [6, 7]. The mpoxv causes an emerging zoonotic disease known as mpox [8]. On November 28, 2022, WHO announced “mpox” as the new name to refer to this re-emerging disease [9]. It is considered to be the most significant opxvinfection in humans, according to the World Health Organization (WHO) [10]. Smallpox and mpox, are both distinct diseases caused by disparate viruses. Smallpox is attributed to the variola virus, is highly contagious, has a higher fatality rate than mpox, does not cause lymphadenopathy, and is eradicated worldwide. Mpox is a self-limiting disease with symptoms lasting from two to four weeks [11]. Currently, three clades of mpoxv are determined: Clade I is present in Congo Basin with up to 10% mortality, clade IIa exists in West Africa with low mortality, and clade IIb is currently spreading globally by human transmission [12].

Poxviruses typically exhibit high environmental stability and great resistance to environmental conditions, consequently, dermal crusts or other materials from infected patients or contaminated fomites might remain infectious for months to years [13]. Outbreaks of mpox frequently affect populations that hunt, kill, handle, and consume bushmeat. The evidence indicates the original entrance of lesion material through the skin, the mucosa, or through a respiratory droplet [14]. Current evidence revealed that mpoxv could be transmitted through sexual intercourse and is considered a potential sexual pathogen [15]. The Mpoxv enters the body through any route (oropharynx, nasopharynx, or intradermal), replicates at the inoculation site, then spreads to nearby lymph nodes. The propagation of the virus and seeding of additional organs follow an initial viremia [16]. The incubation period varies based on the modes of transmission. mpox takes 13 days to incubate when exposed non-invasively (such as through touch with intact skin or droplet transmission), and 9 days when exposed invasively (such as through contact with broken skin or mucous membranes) [17]. The self-limiting nature of mpox typically results in symptoms that last 2–4 weeks. Intense headaches, lesions, fever, and lymphadenopathy are some of the signs and symptoms of mpox. Localized anogenital rashes (with blisters, pustules, or ulcerative lesions) can emerge early and do not spread to other areas of the body, even though oral ulcers continue to be a prevalent feature of fever and lymphadenopathy [18, 19, 20]. The rash often appears one to three days after the fever, though a few patients may experience both at the same time or more than three days later. The most frequent locations for skin lesions are the face (97.5%), torso (92.5%), arms (87.5%), and legs (85%), followed by the genitalia (67.5%), scalp (62.5%), palms (55%), soles of the feet (50%) and the lips (37.5%) [21, 22]. However, the present outbreak has certain atypical characteristics, such as a rash that begins in the genital region and does not spread to other body areas [23, 24]. The symptoms of mpox infection might range from general systemic involvement to include gastrointestinal, respiratory, and other systems. It should be noted that the route of exposure to infection and the quantity of inoculated virus could influence the illness manifestations and severity [17]. The clinical symptoms are characterized by lymphadenopathy, rash, and fever. Lymphadenopathy frequently presents in the groin and neck.

The total number of lesions may range from a few to thousands, and the rash may or may not spread to the rest of the body. The lesions progress at 1-to-2-day intervals through macular, papular, vesicular, and pustular phases throughout the ensuing 2 to 4 weeks. Lesions range in size from 2 to 10 mm, are hard, and undergo synchronous change. Lesions in the genitalia and perianal region, along with the absence of subjective temperature or other prodromal signs, have been present in certain recent cases. Most often, the illness clears up 3–4 weeks after the onset of symptoms, with crusts developing and desquamating during the following 7–14 days. Moreover, pneumonitis, encephalitis, sight-threatening keratitis, and subsequent bacterial infections are some of the possible side effects of mpox disease [16, 25–28]. Mpox is currently a significant threat to the security of the global health system, necessitating the education of patients and healthcare professionals in areas where the mpox is endemic, to improve the development of diagnostic tests, vaccines, antivirals, and other control measures [29]. In this paper, we will review the geographic distribution, transmission, possible reasons for the resurgence of human mpoxv, and prevention strategies.

### Mpox geographical distribution

Mpox has historically been regarded as a sporadic, unusual illness with a constrained ability to transmit between people. These viral disease outbreaks have typically affected communities living in rural areas, small towns (less than 1000 people), humid evergreen tropical forests, or near the human-animal interface [14]. WHO reports that mpoxv is prevalent in 12 endemic countries [30]. There are several countries in Central and West Africa where mpoxv is endemic, including Cameroon, Central African Republic, Cote d’Ivoire, Gabon, Liberia, Nigeria, the Democratic Republic of the Congo (DRC), Sierra Leone, and South Sudan [31]. In 1958, 10 captive monkeys at the Statens Seruminstitut in Copehengan, Denmark, and the Centre d’Enseignement et de Recherches in Paris were found to have the first case of mpox. People who came into contact with infected monkeys were not reported to have contracted any human infections. During this time, there were seven further cases documented [32]. This virus was originally discovered in 1959 as a cause of a pox-like disease in monkeys held at a research facility in Denmark [30]. To conduct cooperation investigations, the WHO took the initiative in 1967. This was done to carry out serological studies, spot mpox outbreaks, and pinpoint the virus’s geographic origins. However, these studies came to no significant conclusions and concluded that mpox is a local illness that cannot spread widely. The mpox outbreak then happened in humans for the first time between 1970 and 1971 [32]. The first human mpox case was reported as a nine-month-old baby who was admitted to the Basankusu Hospital in the DRC on September 1st, 1970 [33]. A total of 59 cases of human mpox were found between 1970 and 1980 in the DRC, Cameroon, Cote d’lvoire, Liberia, Nigeria, and Sierra Leone [34]. Since then, there has been an increase in mpox cases, predominantly noted in the province of the DRC. From 1970 to 1997, around 80% of the occurrences were documented in this area [14]. The WHO described the first 20 human cases between 1972 and 1976, and 15 additional reported cases were added to the 1978 update. Later, between 1970 and 1979, WHO documented 54 cases, of which 47 were detailed by Breman et al. Between 1970 and 1980, Jeek et al. recorded 59 cases [8]. Between 1981 and 1986, the WHO conducted an active surveillance program, reporting a total of 338 confirmed cases and 33 fatalities, a nearly 20-fold increase in reported cases after the surveillance. Between 1993 and 1995, there was a small decrease in the incidence of disease. But soon after, from 1996 to 1997, DRC experienced a significant outbreak [8, 14, 35]. The first epidemic of mpox outside of Africa occurred in the United States (US) in 2003. The index case included a 3-year-old girl who was bitten by an infected prairie dog that was brought to the US from Ghana along with other African rodents. According to the Center for Disease Control (CDC) report, there were a total of 71 cases reported, including both suspected and laboratory-confirmed cases [36–38]. The dry Sudanese savannah was the first place where mpox was discovered in the year 2005. 40 cases in total, both suspected and verified, were noted. When compared to the mpoxv that has historically been reported in the DRC, a change in mpoxv’s genomic structure was seen in this epidemic, demonstrating that mpoxv can adapt to drier locations from humid evergreen tropical forests [8, 39]. In contrast to the fewer than three countries per year in the decades before, six countries reported mpox infected cases in 2017 [40]. In Nigeria, 39 years after the last incidence was documented, human mpox reemerged in September 2017. A suspected case of mpox in an 11-year-old boy was reported to the CDC on September 22, 2017. The boy had an 11-day history of fever, malaise, and the progressive appearance of a vesiculopustular rash on his skin, oral mucosa, and nasal mucosa, as well as associated generalized lymphadenopathy. Following that, the Nigeria Center for Disease Control started a national outbreak response, including increased surveillance for mpox [41]. Since 2017, a significant mpox outbreak with 500 suspected and 200 confirmed cases and a Case Fatality Rate (CFR) of 3% has been recorded in Nigeria. Young children may have a greater CFR than adults [42]. Sporadic cases are still being reported in this nation, frequently with no known epidemiological connections [43]. Two separate importations of mpox from Nigeria to the United Kingdom (UK) occurred in September 2018 [44]. One was a naval officer from Nigeria who traveled to the UK for a training program. The other was a businessman from Nigeria. Both were in good health before the trip, but after arriving in the UK, they both got skin sores. The hospital cleaner who became infected as a result of contaminated bedsheets was the first proven incidence of human-to-human transmission outside of Africa [40]. By June 2019, 165 confirmed cases of mpox had been reported from 17 of Nigeria’s 36 states as a result of increased surveillance in that country [40]. With around 5000 suspected cases in 2019 alone, the DRC reports the most cases of mpox per year in the world [45]. Cases outside of Africa had been documented in the US, the UK, and Singapore before 2022 [31]. Significant global attention has been raised by the mpox outbreak that affected numerous nations in non-endemic regions in 2022 [31]. Over 90,415 confirmed or suspected mpox cases have arisen in at least 106 countries outside of Africa since the virus was first discovered in Europe in early August 2022 [46]. The unexpected onset of the disease in nations including the US, UK, and Singapore in recent years has been related to its African origin [40, 47, 48]. For example, although prior to the global outbreak there had been earlier cases traveling in Nigeria before coming to the UK, the first case to be identified in the UK during the 2022 epidemic was a traveler who had just returned from Nigeria on May 4, 2022 [48]. The WHO declared mpox a “evolving threat of moderate public health concern” on June 23, 2022, as a result of more than 3000 infections with the mpoxv being reported since early May 2022 in more than 50 nations across five regions [20]. Every state in the nation had confirmed cases by November 2022, bringing the total number of cases nationally to close to 30,794. In 114 countries throughout the world, including 107 that had never before reported mpox infections, the outbreak has so far afflicted more than 87,688 people [49].

However, over the past 20 years, several African nations, including the Central African Republic, DRC, Liberia, Cameroon, Sudan, Gabon, Sierra Leone, and Nigeria, have seen an increase in the frequency of reporting and the geographic distribution of cases. Following the eradication of smallpox, there were worries that the mpox may fill the hole in the smallpox epidemiological landscape [40]. Previous experience with smallpox vaccines showed that the immune responces induced by these vaccines have the potential to cross-react with mpox, as there is a high genetic homology (96.3%) between these two viruses [50]. WHO reports that mpox is typically observed in young people under the age of 40 or 50 (varies by country) as a result of the suspension of smallpox immunization following the disease’s elimination in the year 1980. The majority of cases of mpox, with a median age of 31 years, are seen in people under the age of 40 [10]. After the certification of smallpox eradication in 1980, a 5-year period of rigorous surveillance for human mpox in the DRC resulted in the identification and investigation of 338 new illnesses [51]. The mpox outbreak in Germany has so far been concentrated in Berlin and has affected men who have sex with men (MSM) individuals.

Despite outbreaks occurring outside of African nations, human mpoxv is still endemic in this continent [52]. Mpox outbreaks are infrequently documented, poorly controlled, and inadequately described, which results in an imperfect understanding of the disease’s significance. Although mpoxv is the second most pathogenic poxvirus illness after smallpox, it has never received the proper attention to stop it from spreading like an epidemic. It is worth noting that a total of 87,688 confirmed cases of mpox were reported in non-endemic countries throughout the world [49]. Recently, the exportation of mpoxv from Africa to other continents has been progressively reported. However, the lack of travel history to Africa in most of the currently reported cases in 2022 promotes the sign of changing epidemiology of this disease which needs strict epidemiological surveillance to prevent further increase of the recent outbreak in non-endemic countries [49, 53]. Preventing further spread and safeguarding frontline healthcare professionals and others who are most at risk worldwide should be priorities in the current mpoxv outbreak.

### Mpoxv transmission

The mpoxv has received little attention in the past, which has led to a lack of understanding of its transmission pathways [54]. Although the illness name implies that monkeys are the major hosts, the host of the mpox reservoir is unknown, but rodents are thought to be the main host [55]. The mpoxv is primarily spread to people by wild animals like rats and primates, while human-to-human transmission happens frequently [56]. The likelihood of animal-to-human transmission is growing because of environmental conditions increasing the frequency of contact with potential hosts. Living in forested or recently deforested areas, not receiving a smallpox vaccination, handling or consuming dead bush meat or monkeys, and sleeping on the floor in endemic areas, are risk factors for zoonotic transmission of mpox [57]. Transmission from person to person has been connected to respiratory droplets, contact with bodily fluids, contaminated patient surroundings or possessions, and skin lesions on infected people. Large inhaled droplets that are known to disseminate mpox, are unable to move more than a few feet, therefore sustained close contact is necessary for human-to-human transmission [57]. In a review by Beeson A et al., examined key works from animal models, human outbreaks, and case reports emphasized that the evidence for respiratory transmission of mpoxv is limited, but it cannot be ruled out and should be considered in public health recommendations. Mpoxv may be found in droplets like saliva or respiratory secretions that drop out of the air quickly, and there is no evidence for mpoxv being transmitted via airborne particles [58]. The CDC guidance continues to include respiratory-droplet transmission as a way that mpoxv can spread from person to person, and clarifies that respiratory transmission refers to contact with respiratory secretions. While there is no evidence for mpoxv being transmitted via airborne particles, the WHO said that while “short-range” airborne transmission of mpoxv appears to be uncommon, it is possible and warrants precautions [59]. Men who have sex with men (MSM) have been the subjects of the majority of cases so far, especially those who have new or several partners. According to the WHO, transgender and gender diverse people are more susceptible to contracting mpox than other people [60]. The first reports of the 2022 outbreak were from gay, bisexual, and other men who have sex with men (GBMSM) who presented with genital mucosal lesions and reported sexual contact as a possible route of transmission. The rapid evolution of the outbreak is consistent with the transmission in closely connected social and sexual networks of GBMSM, within which high rates of sexual partner change are reported. Travel to large GBMSM events in Spain and Portugal likely increased onward transmission in many countries. A mathematical modeling study found that even a single event of sexually associated mpoxv in a GBMSM population was likely to result in a large outbreak [61]. Most cases have been identified in male MSM particularly, those who have multiple and often anonymous partners. These partners met in places like saunas, cruising bars, and sex clubs, or through dating apps and sex parties [62]. The epidemiology of Sexually transmitted Diseases (STDs) involves a variety of other factors than the number of sex partners. The pattern of mixing between various sexually active groups in the community is one that is significant. Age, place of residence, ethnicity, socioeconomic considerations, or behavioral characteristics, such as the frequency of acquiring sexual partners, may serve as distinguishing characteristics for these groups [63]. More than 30 days after the illness started, DNA can be found in an upper respiratory tract swab, saliva, and semen from recovered patients with Ct values 35 [64]. Estimates of the number of GBMSM at greatest risk of getting and transmitting mpoxv as well as the transmissibility of mpoxv can be made better using clinical and epidemiological data. For the appropriate application of current prevention and control measures as well as the creation of new interventions, for current and future outbreaks, it is imperative to understand the factors that have contributed to the origins and continued pattern of sexual transmission.

Epidemiologic studies show that skin-to-skin and sexual contact, rather than contact with contaminated bedding or clothing, are the main ways that the disease is spread [56]. Moreover, it has been discovered that air travel is a crucial factor in the disease’s spread [65].

#### Virulence as a function of clade

The severity of the disease and the likelihood of human transmission varies between two phylogenetically distinct strains of mpoxv (the Central African clade and the West African clade). The Central African lineage is linked to more severe diseases and spreads more easily through direct contact and massive respiratory droplet transmission. On the other hand, the West African clade discovered to be in charge of the recent outbreak in Nigeria is linked to a milder illness, lower death, and restricted human-to-human transmission [66]. Studies revealed that isolation of diseased people can help prevent the spread of disease [54].

#### Subclinical infection

Few immunological studies that discovered signs of protection against opxvs in asymptomatic people who were exposed to mpox cases are the only ones that have provided evidence of subclinical mpox infection [67–69].

In a retrospective study by Baetselier et al. on 224 men, the presence of replication competent virus in two out of three asymptomatic individuals was reported in Belgium. In the month before and following the sample’s collection, all three males denied experiencing any symptoms. Both they and their contacts did not have clinical mpox, and none of them claimed to have been exposed to a case of the disease. Before that, it was believed that asymptomatic carriership had little impact on the propagation of opxvs. Although the smallpox virus might be found in the upper respiratory tract of asymptomatic contacts of smallpox cases, this virus eradication relied mostly on the detection and isolation of symptomatic cases. Asymptomatic mpox infection is important because it would mean that isolating and identifying symptomatic individuals could not be enough to stop the outbreak if it could spread further. Asymptomatic carriership may contribute more significantly to virus transmission in the ongoing outbreak in non-endemic regions [70, 71]. Outside of endemic areas, the danger of mpoxv transmission in hospital settings is not well known [72].

#### Semen-based transmission

In the current outbreak of disease, mpox transmission through the seminal fluid or sexual activity might be a recognized route. Mpoxv transmission has been documented in the UK in two men with no travel history to endemic countries during sexual intercourse. Furthermore, viral DNA detection in semen samples has been reported in three cases in Italy, two patients with mpox in Germany, and 32 people affected by mpox in a large case series on the 2022 global outbreak [73–75]. In another study prolonged shedding of mpoxv DNA in the semen of infected patients for weeks after symptoms onset supports the transmission of mpoxv during sexual activity [76]. Although quantitative viral DNA detection offers useful data on viral detection and shedding dynamics, more research evaluating viral infectivity is necessary to fully comprehend the potential transmissibility of mpoxv. The viral cell culture is frequently employed as a surrogate for the existence of infectious, replication-competent viruses, and should only be cultured in labs with adequate biosafety facilities [77]. Despite the mild clinical course and low transmission rate, mpox should be viewed in this era of pandemics as a possible risk to public health that necessitates proper containment and study.

### Plausible reasons for the resurgence of the mpox

Emerging infectious illnesses are ones “whose occurrence in humans has increased in the past two decades or threatens to increase in the near future,“ according to the CDC [78]. People have encountered numerous novel viral infectious agents as emerging and re-emerging infectious diseases (EIDs) over the past 20 years. The influence of various factors and elements aids EIDs development. Despite being complicated, these elements can be divided into three categories: ecological, human, and viral factors [79]. Mpox is no longer a rare viral zoonotic disease restricted to isolated regions of Central and West Africa, as evidenced by the recent outbreaks in numerous countries and the spread of the illness across West Africa. Its potential for further regional and global expansion remains a serious concern [80]. There are growing concerns regarding the geographical spread and recurrence of mpox. As of July 26, 2023, it has been reported 30,794confirmed cases and 45 death in U.S [81]. 2933 cases (86%) of the 3413 cases confirmed in 50 countries and territories in June 2022 were reported in European nations. In Nigeria, there was one death reported. In October 2022, 108 nations reported a total of more than 68,000 confirmed cases [82]. A total of 12 confirmed and one probable cases of mpox were reported to the Chicago Department of Public Health between April 17 and May 5, 2023 [83]. Although the exact cause of mpox resurgence is still largely unknown, hypothesized factors including the waning immunity, accumulation of unvaccinated individuals, ecological conditions, risk behaviors of MSM, and genetic evolution are considered as the possible causes of the resurgence in mpox cases [84, 85]. Although the exact mode of transmission of mpoxv is still under investigation, previous research indicated that the majority of virus transmission occurs through animal-to-human transmission and/or through family members [44, 85]. However, most of the transmission in recent cases are human-to-human, demonstrating the susceptibility of the virus to this type of transmission [25, 84, 86]. According to previous outbreaks which occurred almost in rural villages around the forested areas of Africa, the Nigeria outbreak in 2017, and the current outbreak involve people living in the cities. It is plausible and logical that the increase in reported mpox cases is a consequence of increased population density and close contacts, encroachment of human settlements into unknown animal reservoirs, or an increase in the population of susceptible individuals since the cessation of the smallpox vaccination program [84]. According to mpox historical data, mpox outbreaks typically occur in the fall as a result of excessive rainfall, which causes flooding and deforestation, which drive animals, as potential reservoir hosts, toward human populations and residences. Furthermore, the contact between humans and the animal reservoir of mpoxv due to the clearing of forest for new lodging lands, population migration to move deeply in the forest, sleeping outside or on the ground, and living near or visiting the forest were identified as possible factors that increase the risk for exposure to animals and subsequent risk for animal-to-human transmission and resurgence of mpox [87]. Since universal smallpox vaccination programs were discontinued in the 1970s herd immunity has declined over time and a significant proportion of the global population (about 80–96%) does not have immunity against smallpox [84]. It has been demonstrated that in Nigeria, the serologic immunity level against smallpox was 25.7% among vaccinated persons in 2016 and decreased to 9.3% in 2018 [85]. Furthermore, endemic mpox infections were primarily observed among unvaccinated children during the 1970 and 1980s [88, 89]. Therefore, the increase in mpox cases coincides with waning immunity due to the halted smallpox vaccinations, which cross-protected against mpox. The expanding unvaccinated population makes them more susceptible to mpox, increasing the risk of human-to-human transmission [85]. One other plausible factor influencing the resurgence of mpox could be the genetic evolution of the mpoxv [84]. Epidemiological investigations indicated that approximately 90% of confirmed mpox cases had not been infected with other poxviruses, and most cases were born after the end of the smallpox virus eradication program, and very likely have not been vaccinated with the smallpox vaccine. A Phylogenetic study revealed that the mpoxv-2022 strains belong to the same lineage of the mpoxv strain discovered in 2018. However, when compared to the mpoxv strain in 2018, the mpoxv-2022 strains have 46 additional consensus mutations, including 24 nonsynonymous changes [90]. An analysis of the virus genome diversity of 60 samples obtained from humans with mpox infection from Congo revealed four distinct lineages within the Central African clade, as well as a gene loss in 17% of the samples that correlate with human-to-human transmission [91]. In the coding region of mpoxv C9L gene, there is RNA G-quadruplex (RG4) motif that has evolved in different variants. The C9L gene product has a key role in inhibiting the innate immune response of the host. It was shown that evolution declines the stability of RG4 and promotes the C9L protein level. Interestingly, all the evaluated mpoxv genomes during 2022 have had the most unstable RG4 variant, which might be the cause of the raising mpoxv spread [92]. Poxviruses have evolved a unique strategy to rapidly adapt to host antiviral defenses. It was found that poxviruses have “genomic accordions,“ regions of their genome that can expand or contract rapidly in response to selective pressures, such as the host’s immune system. These genomic accordions contain genes that allow the virus to evade host defenses and establish a successful infection. By rapidly expanding and contracting these regions of their genome, poxviruses can quickly adapt to new host environments and overcome host defenses, making them highly successful pathogens. The study sheds light on the evolution and adaptation of poxviruses and provides insights into how viruses can rapidly evolve to evade host defenses [93]. Another recently reported genomic variation, was a strong bias in substitutions of Guanine (G) to Adenine (A) and Cytosine (C) to Thymine (T), which could be due to APOBEC3, a cytidine deaminase. Genomic comparison of mpox from 2015 to 2022 revealed an insertion of a 30-T base length sequence in the middle of the virus genome. However, the role of this variation in virus evolution is not clear yet [92]. Indeed, our understanding of the genetic evolution of mpoxv is still incomplete. Another very important factor that has been evidenced in the recent mpoxv outbreak is the risk behaviors of MSM. Modeling of sexual transmission between men indicates that one-time partnerships, account for roughly 50% of daily mpoxv transmission during the 2022 multinational outbreak of mpoxv in the USA [94]. In the same year in England 82 out of 85 mpoxv confirmed cases had links to transmission in GBMSM sexual networks [95]. These networks ease the human to human transmission which is possibly related to genetic changes [96]. Host adaptation is facilitated by small genetic alterations that are more frequently transmitted at modest rates. Interhuman transmission is supported by stabilizing and destabilizing genetic changes that improve viral fitness and facilitate human to human transmission [97]. More research on mpoxv genomes may allow researchers to observe future adaptations associated with changes in virus properties.

It is worth mentioning that several factors have been implicated in plummeting mpox cases. General factors that may contribute to a decline in the 2022 epidemic cases include: increase education and awareness of the disease, behavior change among the most affected group, increased vaccination efforts, and rising immunity in the sexually active MSM which could limit the virus’s ability to spread. Additionally, the mpox may have burned out as a result of the virus self-limiting, and acquired immunity through natural infection among the GBMSM [98].

#### Mpox prevention

Despite all arduous research efforts, there are no clinically proven treatments for mpox infection. Howevere, Tecovirimat is accessible through CDC-sponsored expanded access Investigational New Drug (IND) protocols as of February 2023 [99]. Therefore, preventative measures including expanding access to mpoxv vaccine and behavioral change effort strategies may be useful in preventing mpox outbreaks [85]. In a recent survey of GBMSM it was determined that they taking actions to protect their sexual health since they learned about the mpox disease. These data highlighted the changing sexual behaviors as follows: reducing the number of sex partners, reducing sex with partners met on dating apps, reducing group sex participation, and reducing attendance at sex venues or social events with close contact [100]. Previous studies have shown that smallpox vaccination provides 85% cross-protection against mpox infection; however, eradicating smallpox and subsequent lack of vaccination efforts paved the way for mpox to gain clinical relevance [85]. Epidemiological investigations revealed that about 90% of confirmed mpox cases had not been infected with other poxviruses, and the majority of cases were born after the end of the smallpox virus eradication program, most likely having not been vaccinated with the smallpox vaccine [57]. Currently, there are three smallpox vaccines: JYNNEOS™, ACAM2000®, and APSV [101]. JYNNEOS™ also known as IMVAMUNE, IMVANEX, MVA-BN is a live, attenuated, non-replicating, and third-generation modified orthopoxvirus vaccine derived from the modified vaccinia Ankara-Bavarian Nordic (MVA-BN strain) which has lost the ability to replicate in mammalian cells. The vaccine is administered by subcutaneous injection as a 2-dose series delivered 28 days apart [92]. JYNNEOS™ was approved by the European Medicine Agency (EMA) and the US Food and Drug Administration (FDA) in September 2019 for the prevention of smallpox and mpox in individuals 18 years of age or who are at high risk of smallpox or mpox infection [93, 101]. JYNNEOS™ does not produce a lesion at the site of vaccination and no longer presents a risk of autoinoculation, inadvertent transmission, or systemic spread [102]. In a UK study, the effectiveness of the MVA-BN vaccine against symptomatic mpox was estimated to be 78% at least 14 days after a single dose in symptomatic GBMSM cases with a rash onset date between July 4 and Oct 9, 2022 [103]. A similar US study among males aged 18–49 years during July 31-September 3, 2022, across 32 U.S. jurisdictions reported a 14-fold higher incidence of mpox disease in unvaccinated people compared with those receiving at least one MVA-BN vaccine dose (equal to 93% vaccine effectiveness) [104]. The adjusted vaccine effectiveness was estimated at 86% by a single dose of subcutaneous MVA-BN vaccine in high-risk male individuals in Israel on 31 July 2022 until 25 December 2022 [105]. In another prominent study from USA between August 15, 2022, and November 19, 2022 the estimated adjusted JYNNEOS vaccine effectiveness was 66.0% for full vaccination and 35.8% for partial vaccination. The findings suggest that a two-dose series of JYNNEOS vaccine provides better protection against mpox disease [106]. ACAM2000® is a live and second-generation vaccinia virus that has been licensed by FDA in August 2007. ACAM2000® is indicated for active immunization against smallpox disease for persons determined to be at high risk for smallpox infection. The CDC allows the use of ACAM2000® during an emergency involving non-variola orthopoxvirus infection including mpox during an outbreak [107]. Vaccination with ACAM2000 is also recommended for laboratory and healthcare workers. The vaccine is produced in primary rabbit kidney cells in tissue cell culture, as such, they have less risk of contamination with adventitious agents. However, it still contains live, replication-competent vaccinia virus and as such is assumed to present the same risk of adverse events [107, 108]. Consequently, guidelines recommend avoiding ACAM2000® among immunosuppressed persons including HIV-infected individuals [109]. APSV (Aventis Pasteur Smallpox Vaccine) is a live and replication-competent vaccinia vaccine [98]. Vaccines used during the eradication campaign included several different strains of vaccinia virus. However, it is not known if this vaccine could be used for mpox [101, 108]. Apart from vaccination, strategies such as surveillance, isolation protocols, and public health interventions can play pivotal roles in curtailing the spread of mpox. Contact tracing is essential in controlling the spread of mpox. Patients with mpox should be interviewed to identify contacts for tracing, including face-to-face contact, direct physical contact, and contact with contaminated fomites. Anyone who has had contact with the patient in the healthcare setting should be identified. If someone is exposed to a person with mpox, they should be monitored for symptoms for 21 days after the last exposure [31]. National surveillance programs are important to control the spread of mpoxv. These programs can provide real-time data on the virus in each affected country, which helps authorities put in place proper public health measures to contain the outbreak [110]. The key objectives of surveillance and case investigation for mpox are to rapidly identify cases, clusters, and the sources of infection as soon as possible in order to provide optimal clinical care, isolate cases to prevent further transmission, identify and manage contacts, and tailor effective control and prevention measures [111]. Since May 2022, countries across Europe, the Americas, and Australia began to report mpox cases in individuals who had no prior travel history to endemic countries. The unexpected appearance of mpox and the wide geographic spread of cases indicate that the mpoxv might have been circulating for some time before the outbreak was detected. The implications of the global distribution of mpox for worldwide public health are significant, and there are attendant risks and concerns. The rising incidence of mpox, particularly in areas with low smallpox vaccination rates, highlights its potential to become a global health threat [112]. The disease disproportionately affects males who have had sex with males. The outbreak of mpox could compound the burden on the health system which already faces multiple other issues. The mpox outbreak is also likely to exacerbate the situation of gender inequality. The isolation of suspected mpoxv cases can lead to domestic violence with women as victims, resulting in job losses and economic instability. Outbreaks will have significant social and political impacts, such as population displacement, increased social tensions, discrimination, and unhealthy competition and protectionism among countries [113].

## Conclusion

Mpox is a viral zoonosis disease having symptoms that were once seen in people who had smallpox. Over time, primary animal-to-human transmission has been the cause of the majority of human infections. Although the specific reason for the resurgence in mpox cases is still mostly unknown, several postulated factors are thought to be contributing to it, including diminishing immunity, an increase in the number of unvaccinated people, ecological conditions, and genetic evolution. For the control of an outbreak, surveillance and quick recognition of new cases are essential. The greatest risk factor for mpox infection occurs during intimate contact with infected individuals during mpox outbreaks. To protect public health, it is crucial to consider all potential modes of transmission and vaccination strategies.

## Data Availability

All data generated or analyzed during this study are included in this article. All authors have read and approved the final version of the manuscript had full access to all of the data in this study and takes complete responsibility for the integrity of the data and the accuracy of the data analysis.
